# From Obesity to Mitochondrial Dysfunction in Peripheral Tissues and in the Central Nervous System

**DOI:** 10.3390/biom15050638

**Published:** 2025-04-29

**Authors:** Francesca Marino, Lidia Petrella, Fabiano Cimmino, Amelia Pizzella, Antonietta Monda, Salvatore Allocca, Roberta Rotondo, Margherita D’Angelo, Nadia Musco, Piera Iommelli, Angela Catapano, Carmela Bagnato, Barbara Paolini, Gina Cavaliere

**Affiliations:** 1 Department of Clinical Medicine and Surgery, University of Naples Federico II, 80131 Naples, Italy; francesca.marino2@gmail.com; 2 Department of Biology, University of Naples Federico II, 80126 Naples, Italy; lidia.petrella@unina.it (L.P.); amelia.pizzella@unina.it (A.P.); angela.catapano@unina.it (A.C.); 3 Department of Human Sciences and Quality of Life Promotion, San Raffaele Telematic University, 00166 Rome, Italy; antonietta.monda@uniroma5.it; 4 Department of Experimental Medicine, University of Campania “Luigi Vanvitelli”, 80131 Naples, Italy; salvatore.allocca1@unicampania.it; 5 Department of Medicine and Surgery, University of Parma, 43126 Parma, Italy; robertarot.97@gmail.com; 6 Department of Biology, Sbarro Institute for Cancer Research and Molecular Medicine, College of Science and Technology, Temple University, Philadelphia, PA 19122, USA; margheritadangelo.dangelo@studenti.unicampania.it; 7 Department of Veterinary Medicine and Animal Production, University of Naples Federico II, 80137 Naples, Italy; nadia.musco@unina.it (N.M.); piera.iommelli@unina.it (P.I.); 8 Clinical Nutrition Unit, Madonna Delle Grazie Hospital, 75100 Matera, Italy; carmela.bagnato@libero.it; 9 Unit of Dietetics and Clinical Nutrition, Department of Innovation, Experimentation and Clinical Research, S. Maria Alle Scotte Hospital, University of Siena, 53100 Siena, Italy; barbara.paolini@unisi.it; 10 Department of Pharmaceutical Sciences, University of Perugia, 06126 Perugia, Italy

**Keywords:** mitochondria, neurodegenerative disorders, obesity, adipose tissue, Alzheimer’s disease, Parkinson’s disease, multiple sclerosis

## Abstract

Obesity is a condition of chronic low-grade inflammation affecting peripheral organs of the body, as well as the central nervous system. The adipose tissue dysfunction occurring under conditions of obesity is a key factor in the onset and progression of a variety of diseases, including neurodegenerative disorders. Mitochondria, key organelles in the production of cellular energy, play an important role in this tissue dysfunction. Numerous studies highlight the close link between obesity and adipocyte mitochondrial dysfunction, resulting in excessive ROS production and adipose tissue inflammation. This inflammation is transmitted systemically, leading to metabolic disorders that also impact the central nervous system, where pro-inflammatory cytokines impair mitochondrial and cellular functions in different areas of the brain, leading to neurodegenerative diseases. To date, several bioactive compounds are able to prevent and/or slow down neurogenerative processes by acting on mitochondrial functions. Among these, some molecules present in the Mediterranean diet, such as polyphenols, carotenoids, and omega-3 PUFAs, exert a protective action due to their antioxidant and anti-inflammatory ability. The aim of this review is to provide an overview of the involvement of adipose tissue dysfunction in the development of neurodegenerative diseases including Alzheimer’s disease, Parkinson’s disease, and multiple sclerosis, emphasizing the central role played by mitochondria, the main actors in the cross-talk between adipose tissue and the central nervous system.

## 1. Introduction

Obesity, one of the most significant public health concerns worldwide [1], is a multifactorial pathology characterized by excessive accumulation of body fat [2], which develops when energy intake exceeds energy expenditure for metabolic and physical activity. The World Health Organization reported that worldwide obesity affects more than 1.9 billion adults, representing 30% of the global population [3]. Caused by genetic, behavioral, societal, and environmental factors, obesity is a strong risk factor for the onset of chronic non-communicable diseases such as type 2 diabetes, cardiovascular illness, various types of cancer, musculoskeletal disorders, and cognitive decline [2].

Obesity, characterized by chronic low-grade inflammation, has been linked to neurodegenerative diseases such as multiple sclerosis (MS), Alzheimer’s disease (AD), and Parkinson’s disease (PD) [4]. Under conditions of obesity, adipose tissue releases pro-inflammatory adipokines/cytokines into the bloodstream [5]. Several pieces of evidence indicate a link between adipose tissue dysfunction, blood–brain barrier (BBB) alteration, neuronal dysfunction, neuroinflammation, and cognitive decline processes. Impairment of BBB integrity leads to increased CNS vulnerability to external factors. In obesity, peripheral inflammatory cytokines invade the CNS, resulting in reduced synaptic plasticity and neurogenesis and promoting the development of neurodegenerative diseases [6]. In this context, mitochondria, organelles that are the main energy source for the cell, play an important role. In fact, numerous studies indicate their involvement in the dysfunction of both adipose tissue and brain tissue in obesity [7]. The aim of this review is to provide an overview of the involvement of adipose tissue dysfunction in the development of some neurodegenerative diseases, such as MS, AD, and PD, under obesity conditions, emphasizing the central role played by mitochondrial dysfunction. In addition, we describe the effects of some bioactive molecules involved in restoring and improving impaired mitochondrial function in neurodegenerative disorders (NDDs).

## 2. Obesity and Inflammation

Obesity, considered as a low-grade chronic inflammation, displays an increased level of inflammatory markers and infiltration of macrophages into white adipose tissue [8]. In obesity, macrophages infiltrating adipose tissue form corona-like structures surrounding adipocytes, leading to the overproduction of adipokines, which comprise pro-inflammatory mediators, such as interleukin-1β (IL-1β), interleukin-6 (IL-6), and tumor necrosis factor-alpha (TNF-α) [9].

Lipid accumulation causes hypertrophy of adipocytes, inducing activation of pro-inflammatory pathways in these cells, such as nuclear factor kappa B (NFκB) [8,10] which is involved in increased production of TNF-α and other pro-inflammatory adipokines. TNF-α is a pro-inflammatory cytokine produced mainly by macrophages and appears to be an important factor in the deregulation of adipokines in adipocytes. Furthermore, it can induce lipolysis, leading to the release of free fatty acids (FFAs). The released fatty acids, in turn, bind to Toll-like receptor 4 (TLR4), which is located on the surface of both adipocytes and macrophages. The binding of fatty acids to TLR4 receptors further activates the NFκB pathway, increasing the production of pro-inflammatory adipokines [11]. Chronic low-grade inflammation in adipose tissue is transmitted systemically, leading to the onset and progression of metabolic disorders [12]. Numerous studies have demonstrated that obesity and related metabolic disorders are conditions that affect not only peripheral tissues but also the central nervous system (CNS) [13].

Adipose tissue dysfunction was correlated with impaired brain metabolism, neuroinflammation, neuronal dysfunction, and cognitive decline [14]. Excessive saturated fat intake and obesity have been shown to reduce the integrity of the blood–brain barrier (BBB), causing brain injury [15]. Specifically, studies in rats fed a Western diet high in saturated fat and sugar showed an increased BBB permeability and increased recruitment of monocytes into the CNS [6]. The structural integrity of the BBB is one of the most critical factors in maintaining brain homeostasis and is regulated by intricate interactions among various cell types, such as endothelial cells, pericytes, and astrocytes [16]. When the BBB is damaged, peripheral inflammatory cytokines and leukocytes migrate into the brain, resulting in reduced synaptic plasticity and impaired neurogenesis [17]. Thus, in obesity, peripheral immune cells can be recruited into the CNS like macrophages in white adipose tissue. This contributes to the activation of microglia, astrocytes, and endothelial cells, resulting in the secretion of pro-inflammatory cytokines such as TNF-α and interleukin-1 beta (IL-1β), which play a key role in the neuroinflammatory process [18].

Saturated fatty acids (SFAs) have been shown activate microglia and trigger the release of pro-inflammatory mediators that can lead to neuronal death [19]. Fatty acids can cross the blood–brain barrier and therefore brain fatty acid levels could be influenced by their presence in the periphery. Therefore, increased levels of circulating fatty acids, as observed in obesity, cause increased brain uptake of SFAs from plasma across the blood–brain barrier, resulting in microglia activation [19]. In vitro studies suggest that SFAs activate microglia to a pro-inflammatory state, increasing the secretion of pro-inflammatory cytokines via Toll-like receptor (TLR)4/NFκB signaling [20]. In vivo experiments have also demonstrated that SFAs activate TLR2 and TLR4 in the rodent hypothalamus, prompting the production of pro-inflammatory cytokines [21]. Furthermore, a high-fat diet compromised neurogenesis in the rodent hippocampus, a brain structure related to cognition, memory, learning, and emotions [22], enhanced lipid peroxidation, and reduced levels of brain-derived neurotrophic factor (BDNF). A growing number of studies have shown that different brain regions, such as the cerebral cortex, hypothalamus, and hippocampus, are subjected to obesity-induced neuroinflammation, which leads to NDDs such as AD [23], PD [24], and MS [25].

## 3. Obesity and Neurodegenerative Disorders

A growing number of data have recently shown that obesity is related to an increased risk of developing neurodegenerative diseases such as AD, PD, and MS, as well as neurological and neurovascular impairments [4,6]. In obese individuals, the risk of developing AD and PD is double that of normal-weight individuals [26,27]. In addition, it was observed that obesity in early childhood and adolescence is a significant risk factor for susceptibility to NDDs [28]. A post-mortem study showed that elderly and morbidly obese individuals have elevated levels of NDD markers [29]. Higher body mass index (BMI) in middle age has been found to predispose to a greater risk of neurological impairment in later life [30].

### 3.1. Alzheimer’s Disease

AD is the most common form of dementia, contributing 60–70% of cases, with the prevalence in women 1.17 times higher than in men [31]. Dementia is currently the seventh leading cause of death and a major cause of disability and dependency in older people worldwide.

AD is an NDD, characterized by progressive neuronal and synaptic loss and biochemical abnormalities, including the deposition of extracellular amyloid beta (Aβ) plaques, tau hyperphosphorylation, and mitochondrial dysfunction, mainly in the cerebral cortex, with a bigger impact on the hippocampus [32]. The initial symptoms of the disease are memory loss, which then advances into various cognitive impairments and eventually leads to death. Several studies reported that obesity has an influence on Aβ deposition. Diet-induced obesity (DIO) is consistently linked to an increase in cerebral Aβ accumulation [33]. Long-term high-fat diet (HFD) consumption in rodents has been shown to result in increased levels of Aβ precursor protein (APP) in both the hippocampus and adipose tissue, combined with an increased inflammatory state. Aβ peptide has been shown to exert functional effects on adipose tissue, leading to increased release of FFAs and pro-inflammatory adipokines [34].

Likewise, a marked association between weight reduction and lower levels of Aβ plaques in the brain has been reported using various dietary regimens, including ketogenic and low-calorie diets [35,36] in different APP transgenic strains, demonstrating that body weight, diet, and obesity have a significant impact on Aβ pathology.

Furthermore, studies have shown that obesity modulates neurofibrillary tangles (NFTs) produced by hyperphosphorylated tau, the microtubule-associated protein [37]. In AD transgenic mice, it was observed that DIO increased Aβ levels and tau phosphorylation. Specifically, an HFD causes Aβ accumulation and tau hyperphosphorylation in the frontal cortex of the 3 × Tg AD mouse model [38].

In addition, in two AD mouse models (3 × TgAD and 5 × FAD), HFD administration is associated with decreased synaptic contacts, signs of cognitive impairment, and oxidative stress in the hippocampus [39].

### 3.2. Parkinson’s Disease

Parkinson’s disease (PD) is the second most common neurodegenerative disease of the CNS after AD. The incidence of PD ranges from 5/100,000 to more than 35/100,000 new cases per year. From the sixth to the ninth decade of life, the incidence increases 5- to 10-fold. PD is characterized by symptoms such as tremor, bradykinesia, muscle rigidity, and impaired posture and balance [40].

A distinctive sign of PD is the degeneration of dopaminergic neurons of the nigrostriatal pathway and the development of intracellular protein aggregates, mainly composed of alpha-synuclein (αSYN), named Lewy bodies, within the neurons [41]. It has been suggested that some of the factors responsible for the degeneration of the dopaminergic system in the CNS, leading to PD, are mitochondrial dysfunction, oxidative stress, and neuroinflammation [42]. Accumulating epidemiological evidence indicates that obesity is a primary risk factor for the development of PD [43].

In obese individuals, adipose tissue has been observed to yield inflammatory adipokines that potentially accelerate disease progression [44]. Evidence shows that saturated FAs are implicated in the pathogenesis of PD, potentially influencing αSYN aggregation, destruction of dopaminergic neurons, oxidative stress, and cytokine production [45]. Studies found that rodents fed with a 45–60% fat diet for 3 to 5 months showed a significant alteration in dopamine receptor expression and dopamine release in the midbrain, with dopamine uptake slowing as of the third month of HFD intake [46]. Furthermore, dopamine receptors and transporters decreased by 42% and 30%, respectively, in rats fed with a high-fat diet for eight weeks [47]. Another study revealed an alteration of dopaminergic neurotransmission after only two weeks of HFD consumption (60%) [48].

### 3.3. Multiple Sclerosis

MS is the most prevalent chronic inflammatory illness of the CNS, affecting over 2.8 million people worldwide. It develops between the ages of 20 and 40 and is more common in young adults and especially women [49].

MS, which affects the brain and spinal cord, is characterized by demyelinating lesions in the white matter of the CNS with axonal degeneration. These lesions cause typical but also unspecific neurological symptoms: difficulties with vision, movement, or balance, leading to disability and reduced quality of life [50]. Recent reports have highlighted that the interaction of environmental and lifestyle risk factors with an individual and susceptible genetic background is implicated in MS development.

In recent decades, risk factors such as obesity, type 2 diabetes, and dyslipidemia have been associated with the onset and progression of MS. Indeed, recent reports showed a correlation between childhood and adolescent obesity and the risk of developing MS [51]. In addition, a study of 8983 patients with MS showed that 31.3% of patients were overweight and 25% were obese [52,53], demonstrating a high incidence of overweight and obesity in the MS population. Furthermore, it has been shown that lipid metabolism disorder is more common in MS subjects compared to normal subjects, with higher levels of oxidized low-density lipoprotein and small high-density lipoproteins [54].

Recently, leptin, an adipokine produced by adipocytes in proportion to the mass of adipose tissue, has been suggested to play a relevant role in the regulation of autoimmune and inflammatory processes and MS disease progression [55]. Leptin is increased in MS patients and adversely correlated with Foxp3 gene expression in PBMCs, which plays a role in suppressing immune function, and is positively related to TNF-α, IL-1β, and hsCRP levels [56]. High levels of leptin may create a positive feedback loop leading to MS progression, as higher levels of this adipokine cause reduced Foxp3 expression and increased production of pro-inflammatory cytokines. These cytokines further reduce Foxp3, which in turn increases leptin and pro-inflammatory cytokines [56].

Recent studies have highlighted that caloric restriction helps to reduce serum leptin levels, and this may increase the survival and lifespan of animal models of inflammation, including those that resemble human MS [57]. Interestingly, calorie restriction reduces inflammation, demyelination, and axon injury without impairing the function of the immune system response [57].

## 4. Obesity, Mitochondrial Dysfunction, and Neurodegenerative Disorders

### 4.1. Obesity and Adipose Tissue Mitochondrial Dysfunction

Mitochondria are double membrane organelles important to the production of ATP in the cell and involved in the adaptation of energy demand, thus playing a relevant role in bioenergetic metabolism. These organelles also represent the main site of cellular production of reactive oxygen species (ROS). Mitochondrial function is closely linked to ROS levels. Under normal conditions, the generated ROS are seized by antioxidant enzymes such as superoxide dismutase, glutathione peroxidase, and catalase [58]. Under conditions of disease or high energy demand, ROS production exceeds the antioxidant capacity of the cell, resulting in oxidative damage. Excessive ROS production is known to damage mtDNA, leading to mitochondrial dysfunction [59]. In diet-induced or genetic mouse models of obesity, decreased OXPHOS capacity and mitochondrial biogenesis were observed in white adipocytes [60,61]. In addition, the number of gene transcripts encoding mitochondrial proteins decreased in obese mice [62], while a decline in mitochondrial mtDNA was observed in obese subjects [63]. Furthermore, both the activity of OXPHOS complexes I to IV and mitochondria’s membrane potential were downregulated in the subcutaneous adipose tissue of obese individuals and in isolated adipocyte mitochondria [64].

Numerous studies have indicated that mitochondrial dysfunction anticipates inflammation in adipose tissue, leading to elevated TNF-α levels, ER stress, and increased ROS in mouse 3T3-L1 pre-adipocytes [65]. Indeed, mitochondrial dysfunction leads to increased ROS generation which activates nuclear factor kappa B, resulting in increased expression of pro-inflammatory cytokines [66] and immune cell infiltration [67,68]. Adipocytes are known to play a significant role in the production of monocyte chemoattractant protein-1 (MCP-1), chemokines that regulate monocytes/macrophage migration and infiltration [10]. It has been observed that mitochondrial dysfunction in adipocytes causes increased expression of MCP-1 [69], which increases macrophage chemotaxis into adipose tissue.

**Figure 1:**
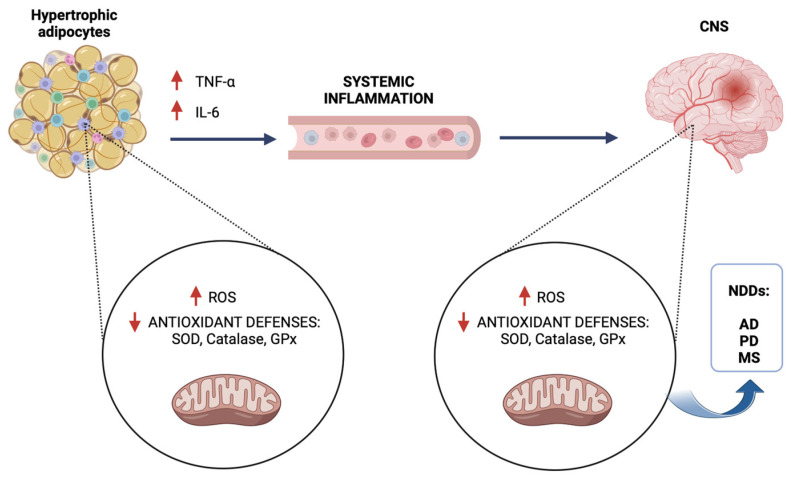
Link between mitochondrial dysfunction in adipose tissue and progression of neurodegenerative diseases. In hypertrophic adipocytes mitochondrial dysfunction leads to increased ROS production, resulting in increased release of pro-inflammatory cytokines, such as tumor necrosis factor alpha (TNF-α) and interleukin-6 (IL-6). The inflammatory state is transmitted via the systemic circulation to the central nervous system (CNS), where it impairs neuronal mitochondrial function and leads to the development of neurodegenerative diseases (NDDs). Alzheimer’s disease (AD), Parkinson’s disease (PD), multiple sclerosis (MS).

Chronic low-grade inflammation caused by mitochondrial dysfunction in adipose tissue is transmitted systemically, leading to metabolic disorders that also impact the CNS, resulting in the initiation and progression of neurodegenerative diseases (Figure 1).

Indeed, numerous studies have demonstrated the relevant role of mitochondria in the development and progression of obesity-induced metabolic disorders in different organs and tissues [61,70,71].

### 4.2. Obesity and Brain Mitochondrial Dysfunction

The CNS, representing only 2% of total body weight, consumes, at rest, about 20% of the inhaled oxygen. Neurons, which are responsible for about 75–80% of the brain energy production, are critically dependent on mitochondrial energy metabolism [72]. It was estimated that there are about two million mitochondria in a neuron [73]. Accordingly, multiple neural functions are supported by mitochondrial ATP, including ion buffering, synaptic vesicle recycling, neurotransmitter synthesis, axonal transport, and the assembly of the actin cytoskeleton [74]. Mitochondria located in the synaptic regions of the neuron play a crucial role in providing ATP for synaptic functions, such as supporting the local system of protein synthesis necessary for synaptic plasticity [75]. Synaptic plasticity is the ability of the nervous system to rapidly adapt to environmental changes, through modification in the number and strength of synaptic contacts during development and in adulthood [75,76,77]. The axonal terminals and dendritic spines are highly plastic sites with enormous energy demands, characterized by a great number of mitochondria. Inhibition of mitochondrial activity has been shown to result in altered synaptic potentiation and compromised neurotransmission, while stimulation of mitochondrial respiration has led to an increase in synaptic density [78]. A reduction in the number of mitochondria is associated with the loss of synapses and spines, as well as a decrease in dendritic length in neuronal cultures [79]. Thus, it is possible to conclude that synaptic functionality and plasticity phenomena depend on mitochondrial activity. Accordingly, mitochondrial dysfunction is directly linked to compromised synaptic plasticity and neuroinflammation [80,81], which are common features of several neuropathologies [82,83]. Nonetheless, it is not yet clear whether mitochondrial dysfunction is a cause or a consequence of inflammatory processes, triggering metabolic adaptations that may lead to protective or detrimental pathways.

Numerous findings correlate mitochondrial dysfunction with different neurodegenerative diseases, such as AD, PD, and MS, but also progressive myoclonic epilepsy type 1 and Friedreich’s ataxia [84,85].

More evidence underscores the impact of dietary fat on brain function and cognitive deficits, indicating that long-term intake of HFDs leads to changes in brain mitochondrial function [51,86]. An HFD has been observed to alter mitochondrial function, leading to reduced oxidative capacity in the cerebral cortex and synaptosomal fraction. Recently, alterations in synaptic plasticity and energy metabolism have been demonstrated in an animal model of diet-induced obesity [80]. An HFD was found to reduce uncoupling in brain mitochondria, resulting in increased production of ROS [80]. Mitochondrial uncoupling is a natural physiological mechanism to limit excessive ROS production. Mitochondria generate ATP via oxidative phosphorylation, but part of the energy produced by electron transport is uncoupled from ATP synthesis due to the proton leak across the inner mitochondrial membrane. This uncoupling allows the mitochondrial membrane potential to be kept below the critical threshold for ROS production. Therefore, ROS generation is minimized by the decrease in mitochondrial coupling [87]. In animal models of diet-induced obesity, increased levels of oxidative stress markers such as MDA and ROS were observed in the hypothalamic region [88] and increased mitochondrial peroxide yield in the hippocampus [89].

In addition, decreased expression of proteins involved in mitochondrial activity was observed in the brains of obese mice, specifically mitochondrial cytochrome c oxidase (COX) subunit 7B (components of complex I and complex IV of the respiratory chain) [90].

In conclusion, loss of neuronal mitochondrial function at the level of synaptic regions impairs neuronal plasticity, as they contribute to the blockage of neurotransmission and cognitive dysfunction associated with neurodegenerative diseases [91]. Evidence correlating mitochondrial dysfunction with major neurodegenerative diseases is given below.

### 4.3. Mitochondrial Dysfunction in Alzheimer’s Disease

AD is a complex pathology in which mitochondrial dysfunction plays a prominent role. Indeed, in AD, mitochondrial dysfunction is responsible for disrupting the normal functioning of synapses, resulting in a reduced neurotransmission and weakened synaptic connections. This impairment contributes to cognitive decline and memory impairment in AD [92]. Accordingly, AD patients display decreased cytochrome c oxidase activity and several deficits in the activity of other mitochondrial enzymes. Moreover, AD neurons show reduced ATP levels and increased ROS levels [93].

A mouse model of AD, overexpressing mutant APP in the brain, shows several alterations at the mitochondrial level. For instance, impaired mitochondrial morphology, increased ROS production, and diminished membrane potential and respiration were observed [94]. It has been proposed that Aβ interferes with mitochondrial function, causing the metabolic deficiencies and neurological dysfunction observed in the brains of AD patients [95]. Moreover, in AD brain and neuronal cultures, Aβ has been shown to disrupt the endoplasmic reticulum–mitochondria connection, resulting in altered mitochondrial morphology, motility, bioenergetics, autophagy, apoptosis, and Ca2+ signaling [96]. In addition, it was observed that mitochondria represent the intracellular site of Aβ accumulation in hippocampal regions of APP transgenic mice [97].

It is well known that excessive/abnormal phosphorylation of tau leads to the formation of insoluble fibers and NFTs, a pathological hallmark of AD, which impair cell function and axonal transport [98]. Studies suggest a nexus between tau and mitochondrial dysfunction. Indeed, an overexpression of tau was observed to induce changes in mitochondrial morphology, mitochondrial transport, and decreased complex I and ATP activity [99]. It has been proposed that tau can be localized in the inner mitochondrial sub-compartments, located between the outer mitochondrial membrane and the inner mitochondrial space, but not in the mitochondrial matrix and could affect the development of contact sites between the ER and the mitochondria [100].

Overexpressed and hyperphosphorylated tau has been shown to impair the localization and distribution of mitochondria, causing defects in axonal function and losses in synapses [101] in several cellular and mouse models of AD. Similarly, alterations in mitochondrial localization have been observed in human AD brains, confirming the link between tau accumulation and mitochondrial translocation [102]. Furthermore, it has been widely observed that the dynamic balance between mitochondrial fission and fusion is disrupted in AD, resulting in a shift toward immoderate fission [103]. Specifically, an abnormal interaction between hyperphosphorylated tau and Drp1, a protein that regulates mitochondrial fission, was observed in brain tissue from 3 ×Tg-AD mice and in brain tissue from AD patients, resulting in excessive mitochondrial fission that induces mitochondrial dysfunction and synaptic degeneration [104].

Long-term mitochondrial fission has also been evidenced in in vitro models of AD. Indeed, overexpression of APP or treatment with Aβ causes severe fragmentation and altered allocation of mitochondria, which likely triggers Aβ-induced synaptic defects in neuronal cultures [105].

### 4.4. Mitochondrial Dysfunction in Parkinson’s Disease

Parkinson’s disease is another neurodegenerative disease in which mitochondrial dysfunction plays a key role and is characterized by the loss of dopaminergic neurons and intracellular protein aggregates, mainly composed of αSYN “Lewy bodies”. The Lewy bodies, a feature of PD, have been demonstrated to accumulate in organelles including mitochondria and both mitochondrial dysfunction and oxidative stress have been linked to PD [106]. Both PD patients and animal models of PD display decreased activities of complex I (CI) of the mitochondrial respiratory chain. Furthermore, partial inhibition of complex I in rat synapses has been shown to increase mitochondrial ROS production [107]. Inhibition of complex I can interfere with energy/ATP generation, causing partial neuronal depolarization attributed to a decrease in Na+/K+-ATPase activity and increased ROS production [108]. In turn, ROS inhibit complex I, creating positive feedback that generates more ROS, increasing oxidative stress and depleting ATP. This alteration of nerve terminal mitochondria has been proposed as a mechanism to explain the neuronal loss in the nigrostriatal pathway that characterizes PD [109]. Therefore, mitochondrial dysfunction has been identified as playing a crucial role in the development of PD.

Neuroinflammation contributes to increased oxidative stress and dopaminergic neuronal loss in the brain of PD patients. Excessive ROS production can cause transcription errors that lead to dysfunction in the expression of several proteins, such as C-terminal αSYN, parkin, and ubiquitin hydrolase, which are directly linked to PD [110]. Mutations in several PARK genes affect mitochondrial function in PD development. Early onset autosomal recessive PD is most commonly caused by mutations in the genes encoding PINK1 (PARK6) and Parkin (PARK2). PINK1 and Parkin serve key roles in mitochondrial quality control mechanisms and the response to mitochondrial damage [111]. PINK1/Parkin has a significant role in the control of mitochondrial fission and fusion and in the induction of mitochondrial biogenesis [112]. The use of Parkin-KO mice in experimental studies showed a decreased mitochondrial respiratory capacity [113]. PINK1-KO mice also showed defects in CI function, alterations in mitochondrial membrane potential, and an increase in mitochondrial fission [114].

Defects in mitochondrial biogenesis were also observed in Parkin-deficient human dopaminergic neurons. Both Parkin and PINK1 mutation or loss in human SH-SY5Y cells result in exacerbated mitochondrial fragmentation mediated by Drp1 [115]. Mutations in the gene encoding for DJ1 (PARK7) are also implicated in PD. Subjects with DJ1 mutations are characterized by early onset and slow progression of Parkinsonism [116]. DJ1 depletion leads to impaired mitochondrial respiration, elevated intracellular ROS levels, impaired mitochondrial membrane potential, and altered mitochondrial morphology [117]. Experimental evidence from isolated mitochondria [118] and rodent tissue [119] underscores how αSYN is a major cause of mitochondrial dysfunction in PD. αSYN has been shown to be a regulator of mitochondria oxidative phosphorylation by interacting with complex I and complex V [120]. Accumulation of oligomers of αSYN or misfolded αSYN in mitochondria reduces complex I activity, ATP synthesis, and organelle biogenesis and increases ROS production, leading to an overall alteration in mitochondrial function and dynamics [121].

Conversely, monomeric αSYN is a physiological regulator of mitochondrial bioenergetics through its ability to interact with ATP synthase and increase its efficiency. Indeed, αSYN knockout mice exhibited lower ATP synthase efficiency, diminished levels of ATP, and an altered neuronal mitochondrial membrane structure and a deficiency of complex I [122].

It has been observed that the interaction of pathological oligomers of αSYN with the alpha subunit of ATP synthase may be mediating aSYN-induced mitochondrial dysfunction [123]. In addition, αSYN oligomers have been seen to interact with protein TOM20, an external mitochondrial membrane protein. As a result of this binding, mitochondrial proteins are compromised and the electron transport chain malfunctions, altering the mitochondrial membrane potential and leading to the accumulation of ROS [124].

### 4.5. Mitochondrial Dysfunction in Multiple Sclerosis

It is well known that the neurodegeneration observed in MS is caused by multiple factors, and mitochondrial dysfunction appears to play a central role, contributing to impaired myelination and axonal damage [125]. N-acetylaspartate is a mitochondrial metabolite and substrate for myelin production by oligodendrocytes (after decomposition into acetate and aspartate). Decreased N-acetylaspartate uptake from dysfunctional mitochondria lead to the low myelination observed in MS patients [126].

The reduction of myelin observed in MS is followed by an increase in the energy requirements of the axon to maintain its resting membrane potential [127]. Neurons and oligodendrocytes (OLs) in post-mortem MS tissues and animal models of MS have shown alteration of normal mitochondrial functions [127]. Accordingly, a decreased expression of mitochondrial complexes was observed in upper cortical motor neurons of MS patients [128]. Several studies revealed that inflammation-related mitochondrial damage in MS is linked to the blockade of the mitochondrial chain complex IV [129], a pivotal component of mitochondrial activity responsible for approximately 90% of total cellular oxygen consumption. An increased activity of complex IV has been demonstrated in chronic demyelinating lesions [130], suggesting that this complex may play a crucial role in counteracting the energetic consequences of inflammation-related axonal demyelination. Nitric oxide (NO), a mediator released during inflammatory demyelination, and related species could be detrimental to mitochondrial complex IV because they can compete with molecular oxygen (O_2_) in binding the functional site of COX, the main subunit of the complex, resulting in transient inhibition and reversible conduction block in axons [131]. Overproduction of NO is one of the features of neuroinflammation-related CNS diseases, including MS. The role of pro-inflammatory cytokines in NO production is significant in both the peripheral immune system and the CNS [132].

Furthermore, a decrease in PGC-1 levels and in the expression of various mitochondrial protein components in pyramidal neurons has been observed in patients with MS [133]. In addition, higher levels of a heat shock protein (mtHSP70), a marker of mitochondrial stress, were found in MS subjects [134]. Moreover, an enhanced mitophagy was detected in neurons of MS patients, although it is not clear whether this process is associated with pathogenesis or represents a compensatory mechanism [135].

In mitochondria-enriched fractions isolated from the brain cortex of post-mortem MS patients, a reduction in mitochondrial electron transport chain gene expression was observed but no deficiency in the number of mitochondria themselves [128]. In addition, complexes I and III were found to be decreased by 61 and 40%, respectively (compared to control), but there were no variations in complex IV [128]. Several studies have been conducted using animal models of MS, such as experimental autoimmune encephalomyelitis (EAE) mice and cuprizone (CPZ)-induced demyelination mouse models.

In animal models of MS, demyelination has been shown to have a direct impact on mitochondrial dynamics through changes in fission and fusion. Specifically, an increase in DRP1 expression was observed in the spike-injured spinal cords of EAE mice and in the corpus callosum of CPZ mice. While inhibiting Drp1 activation, a neuroprotective effect has been found in both EAE and CPZ [136]. In addition, an enhanced Mfn2 expression was observed in proteolipid (PLP)4e mice, a demyelinating mouse model containing extra copies of myelin genes [137].

## 5. The Role of Bioactive Compounds in Obesity and Neurodegenerative Disorders: Focus on Mitochondrial Function

**Figure 2:**
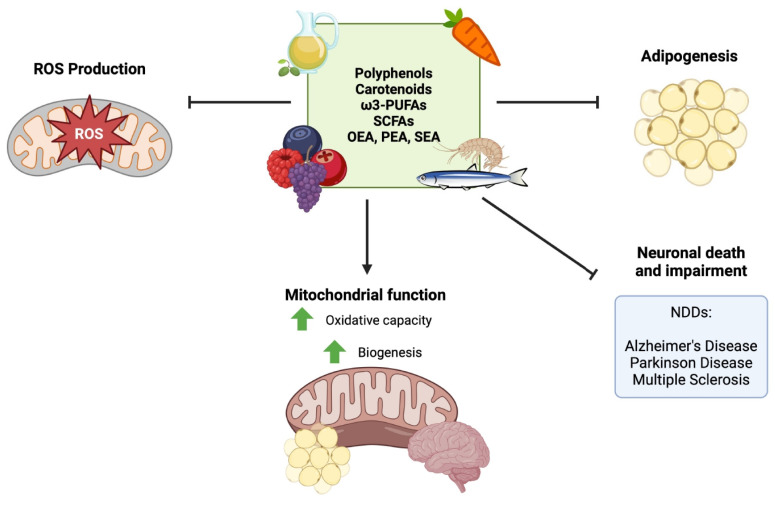
Role of biomolecules in preventing or reversing obesity and neurodegenerative disorders. Polyphenols, carotenoids, ω-3 polyunsaturated fatty acids (ω-3 PUFAs), short-chain fatty acids (SCFAs), and endocannabinoid-like compounds (oleoylethanolamide (OEA), palmitoylethanolamide (PEA), and stearoylethanolamide (SEA)) prevent or reverse obesity and neurodegenerative disorders (NDDs) by counteracting adipogenesis, ROS production, and neuronal death and by increasing mitochondrial function.

To date, many studies conducted both in vivo and in vitro have demonstrated the important role played by some biomolecules, such as polyphenols, carotenoids, fatty acids, and endocannabinoid-like compounds, in the prevention of or recovery from obesity and NDDs (Figure 2). The studies reported below illustrate the biological effects of the administration of pure molecules in vivo or in vitro experimental models.

### 5.1. Polyphenols

Polyphenols, classified as secondary plant metabolites, are known for their antioxidant properties and cytoprotective and anti-inflammatory effects [138].

Resveratrol (RES, 3, 5, 4′-trihydroxystilbene) and curcumin (CM) have been proven to have cytoprotective effects, preventing and/or minimizing cellular and mitochondrial damage. In addition, these compounds exhibited both anti-lipid effects and relevant neuroprotective activities, among many other beneficial properties. In general, the neuroprotective benefits of a diet rich in polyphenols have been studied and confirmed by numerous studies in cell and animal models [139].

RES is a phenolic micronutrient compound, which occurs naturally in blueberry, grapes, mulberry, and other fruits. The precise mechanism by which RES achieves its beneficial effects are not yet fully understood. Several recent pieces of evidence have demonstrated that it performs its action through mitochondria modulation, proving to be a substance capable of enhancing their functions [140]. The inhibitory effect of RES on adipogenesis has been examined by several studies conducted in vitro and in animal models. In pre-adipocyte cells incubated with increasing concentrations of RES (1, 10, and 25 µM) for 24 h, a decrease in triacylglycerol content and decreased expression of acetyl-CoA carboxylase (ACC), the first enzyme with a key role in the synthesis of long-chain saturated fatty acids, were found [141]. In an animal model of obese Zucker rats, a reduction in abdominal fat and ACC enzyme activities was found after administration of RES 10 mg/kg for 8 weeks [142]. RES would hinder the process of adipogenesis by activating the AMPK signaling pathway [143], an energy sensor responsible for metabolic modifications by phosphorylating downstream substrates, such as ACC. RES exert an anti-adipogenic effect by promoting the expression of genes involved in oxidative phosphorylation and mitochondrial biogenesis through stimulation of SIRT-1 deacetylase [144]. Moreover, studies have demonstrated the ability of RES to prevent cognitive impairment and neurodegeneration through its antioxidant and anti-inflammatory properties. RES enhances the level of cellular antioxidants, such as glutathione, superoxide dismutase, glutathione peroxidase, and catalase, and reduces lipid damage due to the peroxidation events [145] in brain tissue. RES has been shown to decrease superoxide radical synthesis by stimulating complex III activity and COXIV activity [146].

Reports suggest that RES can be used to treat and manage AD, exerting a protective action against Aβ-induced neuronal oxidative damage [147,148]. RES ameliorates motor dysfunction in the A53T αSYN mouse model of PD and reduces striatal αSYN levels, glial activation, and TNF-α and IL-1β levels in an MPTP mouse model of PD [149]. RES improves brain mitochondrial health by modulating the activation and expression of peroxisome proliferator-activated receptor coactivator-1α (PGC-1α), which is liable for mitochondrial biogenesis [149], boosting mitochondrial mass.

CM is a polyphenol derived from Curcuma longa L. and is known to exert multiple beneficial effects including weight loss and reduction in the incidence of obesity-related diseases [150]. CM inhibits adipocyte differentiation, promotes antioxidant activities, and prevents macrophage infiltration and activation of nuclear factor κB (NFκB) in adipose tissue [151]. Moreover, CM was observed to remarkably improve mitochondrial respiratory function in mature adipocytes, specifically accelerating mitochondrial basal respiration, ATP production, and expression of mitochondrial uncoupling protein 1 (UCP1). CM performs these actions via the regulation of peroxisome proliferator-activated receptor-γ (PPAR-γ) in 3T3-L1 adipocytes and in an obese rodent model [152]. In addition, studies have shown its beneficial therapeutic properties in CNS cells, particularly in attenuating mitochondrial dysfunction. CM has been shown to improve neuronal survival by reducing apoptosis, oxidative stress, and neuroinflammation via activation of the CREB-BDNF pathway [153]. CM has been observed to act by activating Nrf2 and Nrf2 target genes in primary astrocytes, thereby reducing intracellular ROS levels and attenuating oxidative damage and mitochondrial dysfunction. CM restores mitochondrial function through induction of the nuclear receptor PGC1α, showing itself as a hopeful agent that can delay brain aging and forestall mitochondrial damage [154].

### 5.2. Carotenoids

Carotenoids (CTs) are substances corresponding to a large family of C_40_ lipophilic pigments produced by photosynthetic organisms and some prokaryotes and fungi. They play an important role as antioxidants, primarily as lipid defenders against the damaging effects of oxidative stress.

CTs have been shown to have a significant impact on the treatment of obesity and neurodegenerative diseases [155]. They perform their function by maintaining mitochondrial function and integrity [156]. Studies have been conducted to examine the potential use of CTs in the managing of obesity. In particular, the effects of pure carotenoid supplementation on obesity have been investigated in two randomized double-blind placebo-controlled clinical trials. In the first, a reduction in BMI, waist-to-height ratio, and subcutaneous adipose tissue was observed in children who received a carotenoid mixture for 6 months [157]. In the second, a mixture of CTs was given to healthy overweight volunteers for 12 weeks, resulting in a reduction in total fat area and BMI compared with a placebo group [158]. CTs have been observed to act by inhibiting adipocyte differentiation by interfering with nuclear receptors like RAR, RXR, or PPAR [159]. Studies suggest that the brain is involved in body weight regulation under the influence of CTs. These compounds have been found in various regions of the adult brain [160]. Interestingly, it has been suggested that these compounds would cross the blood–brain barrier and affect neurons in the arcuate nucleus, influencing feeding behavior and preventing weight gain and adiposity [161].

CTs are considered beneficial in the treatment of NDDs, exhibiting anti-neuroinflammatory properties and a significant role in the prevention of PD and AD. Indeed, CT supplementation has been shown to be effective in hindering αSYN toxicity and protecting against the amyloid and tau diseases [155,162]. These beneficial effects are due to their ability to improve mitochondrial function. In support of this, it has been shown that deficiency of the enzyme β-carotene oxygenase 2 (BCO2), which is involved in the catalytic activities of CTs, leads to superoxide overproduction in mitochondria and decreases the level of mitochondrial superoxide dismutase, affecting mitochondrial respiratory complexes [163].

Lycopene (LYC) is the main carotenoid present in tomatoes and tomato products, and its anti-obesity effect has been shown in mice fed a high-fat diet, whose adiposity was reduced after supplementation. LYC could reduce body weight gain by increasing energy expenditure. Indeed, LYC was found to increase mitochondrial biogenesis and function in obese mice by increasing basal mitochondrial respiratory capacity and ATP-linked respiration. Furthermore, LYC increases PGC-1α and UCP1 expression in mature adipocytes and adipose tissue [164]. LYC would exert its action by downregulating lipogenesis genes and upregulating those related to lipidolysis, such as thermogenic and mitochondrial functional genes [165]. Recent advances have demonstrated a neuroprotective effect of LYC. In particular, it improves cognitive abilities and alleviates memory impairments in tau transgenic mice, an animal model of AD [166]. In addition, this compound decreases the secretion of Aβ, downregulates amyloid precursor protein (APP) levels, and decreases tau hyperphosphorylation in the brain [166]. LYC has been reported to restore mitochondrial dysfunction in AD and PD through its antioxidant and anti-inflammatory properties. Specifically, it can prevent Ab-induced cell damage by activating the PI3K/Akt/Nrf2 pathway [91,167,168].

Astaxanthin (ASX), also called 3,3′-dihydroxy-β, β′-carotene-4,4′-dione, is a carotenoid with a bright orange to red color found in marine animals such as shrimp, lobster, crab, and salmon and some other organisms such as algae. In terms of antioxidant activity, ASX is ten times more potent than other CTs and 100 times more potent than α-tocopherol [169]. ASX has been shown to improve obesity and its associated conditions. Indeed, its supplementation and physical training over a 12-week period in a group of obese men led to a reduction in adiponectin levels and body weight and fat percentage and improved lipid and metabolic profiles. In addition, ASX has been observed to inhibit body weight gain and lipid accumulation and reduce the release of pro-inflammatory cytokines in mice fed a high-fat diet [170]. This is due to its ability to activate AMPK and upregulate the expression of transcription factors involved in mitochondrial remodeling and increased mitochondrial component of oxidative phosphorylation [171]. Furthermore, ASX has been reported to contribute to the enhancement and maintenance of mitochondrial activity, promote mitochondrial biogenesis, and inhibit mitochondrial fission/fragmentation by activating antioxidant and anti-inflammatory pathways in various cell lines [172]. ASX acts by inhibiting canonical NFκB signaling in response to oxidative stress. Specifically, it suppresses NFκB-mediated gene expression of pro-inflammatory cytokines such as IL-1β, IL-6, or TNF-α, thereby inhibiting the development of inflammation. Current studies show that ASX has a preventive effect on oxidative-stress-induced neurodegenerative pathological conditions by maintaining the integrity of the BBB and ameliorating neuroinflammation induced by the neurotoxin or stressful conditions such as glucose deprivation/reperfusion treatment in the rodent brain [173]. Specifically, ASX acts by preserving the stability of tight junctions of microvascular endothelial cells [174]. In a mouse model of AD, ASX in the ester form with docosahexaenoic acid decreases oxidative stress and inflammasome activation [175].

### 5.3. Fatty Acids

After adipose tissue, the brain is the organ with the highest amount of lipids, with approximately 50% of its dry weight being lipids. Lipids play a crucial role in the normal development and function of the CNS. The type of FAs incorporated into the phospholipids of brain cell membranes determines membrane structure, microdomain organization, and fluidity. Data have shown the impact of dietary lipids on the degree of lipid accumulation in the body, as well as the structure and functioning of the brain [176]. Accordingly, alterations in cholesterol metabolism have been implicated in the pathogenesis of several NDDs [177]. On the other hand, numerous studies clearly demonstrate that consumption of omega-3 polyunsaturated fatty acids (PUFAs), such as docosahexaenoic acid (DHA) and eicosapentaenoic acid (EPA), attenuates diet-induced obesity by increasing mitochondrial lipid oxidation and reducing energy efficiency through modulation of the mitochondrial uncoupling [178,179]. In addition, omega-3 PUFAs, particularly DHA, can affect brain function [180]. Indeed, a positive association between high DHA consumption or high serum DHA levels and a reduced risk of developing AD was observed in a population of patients aged 65 to 94 years. In addition, DHA demonstrated a protective effect against typical AD pathological mechanisms [181]. Accordingly, recent prospective questionnaire-based studies have reported that a high intake of unsaturated fatty acids may protect against PD [182].

It is well known that PUFAs have important effects on phospholipid composition of mitochondrial membranes and on the functionality of these organelles, as shown in experiments conducted on animal models of aging, AD, and PD, which exhibited an improvement in brain mitochondrial function after treatment with PUFAs such as DHA [183,184]. Specifically, ω-3 PUFAs exert neuroprotective effects by counteracting oxidative stress and consequent alterations in the structure and function of mitochondrial transporters and enzymes involved in ATP production and glucose metabolism. Different studies confirm the beneficial effects of omega-3 PUFAs on inflammatory parameters by modulating mitochondrial respiratory parameters in the liver, skeletal muscle, and brain through supplementation intake in animal models [185,186,187].

Conjugated linoleic acids (CLAs) are a class of isomers of linoleic acid, known to exhibit important beneficial effects on human health. Several studies have shown their ability to counteract HFD-induced obesity [155,188,189]. CLAs have been observed to activate several nuclear receptors involved in the regulation of gene expression related to lipid metabolism. Specifically, CLAs decrease lipid synthesis, adipogenesis, and lipid storage in adipocytes and increase β-oxidation in skeletal muscle [190]. Furthermore, the trans-10, cis-12 isomers have been shown to influence body composition, decreasing adiposity by promoting the browning of adipocytes in mice [191]. In addition, the preventive effect of CLA supplementation against age-dependent neurodegeneration in the mouse model has been demonstrated, likely due to its effect in reducing oxidative stress and improving mitochondrial function, also noted in various organs and tissues [192,193]. This protective effect may occur through the activation of nuclear factor erythroid 2-related factor (Nrf2), which plays a key role in the preservation of redox homeostasis and mitochondrial function and preventing the inflammatory state [194].

Short-chain fatty acids (SCFAs, microbial metabolites such as butyrate, propionate, and acetate) have been shown to have many beneficial effects on human health, including an anti-obesity effect [195]. In fact, human studies have demonstrated that administration of SCFA to obese patients resulted in the increased production and secretion of the satiety-promoting hormone peptide YY (PYY) and glucagon-like peptide-1 (GLP-1), with consequent reduction in adiposity [196]. Furthermore, studies in animal models have shown that butyrate can counteract obesity by increasing mitochondrial uncoupling and thereby promoting inefficient metabolism [197]. Butyrate is a promising candidate for the treatment of NDDs [198]. Indeed, different studies have shown that it has a neuroprotective effect in PD mice through indirect modulation of the gut microbiota, preventing inflammation in the gut–brain axis [199,200]. Moreover, by improving mitochondrial functions, butyrate counteracts the inflammatory processes and oxidative stress induced by an HFD in the cortex and synaptic area of the mouse brain. Thus, it may be involved in regulating neural plasticity and preventing the development of NDDs [200,201].

### 5.4. Endocannabinoid-like Compounds

In recent years, numerous researchers focused their attention on molecules with an endocannabinoid-like structure, known as endocannabinoid-like compounds, such as palmitoylethanolamide (PEA), oleoylethanolamide (OEA), and stearoyl ethanolamide (SEA), which have demonstrated both an anti-obesity effect and the ability to prevent the development of NDD. Despite numerous studies, the molecular mechanisms underlying these effects are still not fully understood. They are present in small quantities in food, but their main origin is endogenous synthesis [202].

PEA is an endogenous lipid mediator belonging to the N-acylethanolamine family, widely studied for its pleiotropic effects both centrally and peripherally. Several pre-clinical in vitro and in vivo studies indicate that PEA, an element naturally contained in some foods, can counteract obesity and be a potent agent for effective therapy of NDDs [203]. PEA reduced food intake, body weight, and body fat mass, effects mediated in part by restoring central and peripheral sensitivity to leptin in rats [204]. It has been observed that PEA stimulates the beige-conversion of the subcutaneous white adipose tissue (WAT) by increasing thermogenic markers and by PPAR-α activation. In addition, PEA can improve mitochondrial bioenergetics in mature adipocytes via AMPK phosphorylation [205]. It has been suggested that PEA could be a potential therapeutic agent for obesity-related neuropsychiatric comorbidities by regulating neuroinflammation, BBB breakdown, and neurotransmitter imbalance related to behavioral dysfunction [206]. Due to its antioxidant capabilities, PEA has been seen to enhance mitochondrial function in hippocampal regions and other tissues [207,208]. It also exerts its beneficial effects on neuroinflammation by acting on the composition of the gut microbiota, involving the microbiota–gut–brain axis [207].

OEA, an endocannabinoid-like compound, has been shown to have protective effects in many metabolic disorders by modulating the expression of genes involved in feeding behavior and lipid metabolism regulation [209]. Indeed, in rodents, intraperitoneal administration of OEA induces satiety, weight loss, stimulation of lipolysis, and enhancement of fatty acid oxidation by increasing the gene expression of proliferator-activated receptor-α (PPAR-α), a major player in the regulation of intra- and extracellular lipid metabolism [202,209]. Therefore, it is suggested as a potential therapeutic agent for the treatment of obesity. In addition, accumulating evidence has shown the protective role of OEA in inflammatory processes and NDDs. Indeed, it is an anti-inflammatory agent able to attenuate Aβ pathology in a mouse model of AD by modulating lipid metabolism and microglial phagocytosis [210]. OEA-mediated neuroprotection has been tested on in vivo and in vitro models of 6-hydroxydopamine (6-OH-DA)-induced degeneration, which causes Parkinsonian symptoms. In this animal model, administration of OEA prevents or alleviates Parkinsonian symptoms [211]. It was also tested against mitochondrial toxicity due to the electron transport chain complex II inhibitor, 3-nitropropionic acid (3-NP), in rat cortices, showing its ability to preserve mitochondrial functional integrity and cell viability [212]. It is therefore considered a potential agent for the treatment of NDDs.

SEA is a cannabinoid-like compound with a wide range of biological activities. It has anti-inflammatory properties that have been demonstrated in various animal models of pathological conditions. However, the molecular mechanisms are still unclear. Several studies have demonstrated the anorectic capacity of SEA in the mouse animal model. In addition, this anorectic effect is accompanied by a reduction in the mRNA expression of hepatic stearoyl-CoA desaturase-1 (SCD-1), which has recently been proposed as a molecular target against obesity. This suggests that SEA could have important implications in the pharmacological treatment of obesity [213]. SEA administration also had a positive impact on restoring the FFA composition of adipocytes taken from rats of different ages with HFD-induced insulin resistance, demonstrating its potential as a treatment for obesity-related complications [214]. In addition, SEA showed a neuroprotective effect against LPS-induced neuroinflammation in male C57BL/6 mice. In particular, by supporting blood–brain barrier integrity, SEA limited the spread of peripheral inflammation to the brain, thereby preventing the activation of resident microglia and the trafficking of leukocytes towards the brain parenchyma. Therefore, SEA can also be considered as a candidate for preventive therapy of cognitive dysfunction caused by neuroinflammation [215].

## 6. Conclusions

In recent years, a wealth of data has highlighted the correlation between obesity and NDDs. Although the exact underlying mechanisms remain unclear, it has been hypothesized that the adipose tissue dysfunction that occurs in obesity leads to systemic inflammation and to the alteration of the BBB resulting in neuroinflammation and cognitive decline. In this scenario, mitochondrial dysfunction plays a crucial role. Therefore, bioactive compounds that enhance mitochondrial function and eliminate excess ROS can be used to treat NDDs.

This review has discussed the beneficial effects of some bioactive food components in restoring and improving CNS mitochondrial function, supporting the importance of a healthy diet in preventing or slowing the onset and progression of NDDs. Among the bioactive compounds mentioned, polyphenols, CTs, and omega-3 PUFAs play an important role due to their antioxidant and anti-inflammatory effects, which can prevent ROS damage and improve mitochondrial function.

However, further studies are needed to define the exact molecular pathways underlying the onset and progression of NDDs and to elucidate the mechanisms driving the beneficial effects exerted by bioactive molecules in the treatment of mitochondrial dysfunction in NDDs. The results of these studies will allow setting up novel pharmacological approaches aimed at preventing the devastating progression of the aforementioned diseases. Therefore, this review encourages researchers to focus their attention on mitochondrial dysfunction in the development not only of NDDs but of all obesity-related pathological conditions.

## Data Availability

Not applicable.
